# Association American Medical Editors

**Published:** 1886-05

**Authors:** 


The Asssociation of American Medical Editors held their annual meeting in St. Louis, May 3d, and were entertained at dinner by the "Press and Library Club." The officers for the present term are, president, John V. Shoemaker, M. D., of the Pennsylvania *Medical Bulletin*; vice-president, D. S. Reynolds, M. D., of the Louisville *Progress*; secretary, William Porter, M. D., president of the St. Louis Press Association.

